# No Coming Back to Sick Society: The Emergence of New Drug User Segment in the Järvenpää Social Hospital in Finland, 1965–1975

**DOI:** 10.1093/jhmas/jrab038

**Published:** 2021-09-21

**Authors:** Katariina Parhi

**Affiliations:** Tampere University, Finland

**Keywords:** drugs, psychiatry, history of medicine, social work, Finland

## Abstract

This article explores the emergence of “new” drug users by taking a closer look at the medical records of individuals who received inpatient treatment in the Järvenpää Social Hospital in Finland due to their drug consumption during the years 1965–1975. The hospital focused on social and psychiatric care. The Social Hospital patients differentiated themselves between “classical narcomaniacs” and new users, which indicates that a new user segment had emerged. The hospital staff described the new patients’ personalities as shortsighted and dependent on others but avoided making homogenic or dogmatic psychiatric interpretations. Both the patients and the staff referred to “drug philosophy” or “drug ideology,” which positioned drug consumption in a certain work-avoiding societal context. The main argument is that the Social Hospital archive provides an invaluable source by offering a combination of medical and societal analysis together with patients’ perspectives. Drug use was seen as a social problem that was never simplified into any one cause. Instead, the files offer a nuanced view to drug use and youth culture in Finland.

## Introduction

In 1961, Finland became the second Nordic country – after Norway – to include treatment of drug users within the same legislation as treatment of alcoholism. Since the 1930s, there had been a national alcohol treatment system in Finland, which was based on strict social control and legal procedures. The system came to include drug treatment in the 1960s.^1^ Internationally, drug use had become more common between the 1880s and 1920s. This increase was related to the professionalization of pharmacology, new standardized anesthetics and painkillers, an increase in the number and use of syringes, and the development of patent medicines and the pharmaceutical industry.^2^ For a long time, drug use remained marginal in Finland. Until the 1950s, morphine was the most commonly abused substance, whereas cocaine and opium were less frequent.^3^ During the Second World War, heroin use became more common. Abuse of prescription medicines was also common, though less scandalous – which was not unique to Finland. For example, in France, the discourse around some drugs, such as LSD and marijuana, became more accusatory than around the abuse of prescription medicine. The moral divide exemplifies how the boundary between illicit drugs and pharmaceutical medicines has been artificial.^4^ There are also boundaries between use and abuse – the meanings are aligned with different culturally rooted calculations.^5^ The Finnish 1961 Act on the Treatment and Care of Abusers of Intoxicating Substances (*Laki päihdyttävien aineiden väärinkäyttäjien huollosta* 96/1961) combined the treatment of alcoholics and drug users by using the term “intoxication” as a common denominator. In addition to the new emphasis on drugs, the law also paid special attention to users between 18–24 years of age, although they could not be institutionalized against their will.

The historian Johan Edman and social work scholar Kerstin Stenius portray the 1961 law as a long process. Drugs became a political problem after WWII, and the conceptualizations for the causes of use were at the time “vague and non-contested.” Although their use was only marginal, the drug problem was constructed primarily as a youth problem, which was a potential threat to the future of the state and its citizens. This view became a strong argument for paternalistically motivated coercive interventions.^6^

The drug scene changed dramatically in the 1960s. The sociologist Pekka Hakkarainen has listed some characteristics of the new drug scene of the 1960s in Finland: cannabis was the most typical substance. The number of users expanded, and the users were younger than before. The focus went from addiction to experimentation with drugs, with the emphasis on psychoactive drugs, expanding consciousness, and the symbolic meanings of use. New users became increasingly dependent on smuggling in comparison to substances that were available in pharmacies.^7^ The drug issue was most prevalent in the Helsinki area. By the end of the 1960s, treatment facilities and other forms of help were scattered around Helsinki and run by over twenty-five different organizations, such as mental hospitals, societies, A-Clinic, and The Mannerheim League for Child Welfare.^8^ The treatment facilities were scarce and unorganized. The only physician-led institution for alcoholics had been renamed *Järvenpään sosiaalisairaala* (Järvenpää Social Hospital)^9^ in 1962. The name change of the hospital was a result of the 1961 law^10^ – the previous name *Alkoholistien vastaanottolaitos* translates as a receiving facility of alcoholics, which excluded other intoxicants. The new name referred to a unique positioning between social and mental care. The Social Hospital was not a mental hospital, as it did not treat psychosis, but it was not targeted at mere social problems either, as it offered medical and psychiatric help.^11^

This article takes a closer look at the records of patients who received inpatient treatment in the Social Hospital in Finland due to their drug consumption during 1965–1975.^12^ The focus is on the emergence of a “new” drug user segment. Although the law changed in 1961, the ten-year period between 1965 and 1975 is seen as a significant peak in drug consumption, referred to as the “first tide of drugs” in Finnish drug historiography.^13^ The first section begins by introducing some of the problems in historiography in relation to drugs and their use, and then moves on to discuss what the Social Hospital records can add to the knowledge of drug use in regards to the time period of 1965–1975. The second section exemplifies how the Social Hospital patients differentiated themselves between “classical” and “new” users. This division is significant because it shows that a new user segment had emerged. The third section shifts the focus to the ways the Social Hospital staff perceived their patients, offering a more medicalized perspective, albeit in a hospital that emphasized the social aspects of intoxication. The fourth section links drug consumption more strongly to the societal context, both nationally and internationally, and discusses “drug philosophy” and “drug ideology” as described in the sources. The basic idea behind such philosophies and ideologies was the avoidance of work. One of the aims of this article, following the formulation of the media sociologist, Paul Manning, is to study the interplay between representations and actual drug use. Manning describes this interplay as a “shared symbolic universe” – intersections of media representations and drug consumption at the micro-level.^14^ These intersections should also include the influence of the treatment facilities and medical knowledge, as these facilities were an essential part of drug-related life for many. Medical records help us to see what drug-related problems looked like in institutionalized settings. Institutions took part in building drug user identities during an era when these identities were only just taking shape. I argue that the Social Hospital archive provides an invaluable source by offering a combination of medical and societal analysis together with patients’ perspectives. Drug use was a highly politicized topic at the time and the debates on drugs in Finnish society were heated and emotional. Despite opinionated arguments, drug use in Finland was foreign to most debaters. Archival sources present drug use in the 1960s and 1970s as a social problem that was never simplified into any one cause. Instead, the files analyze various diagnoses and societal issues, offering a nuanced view to drug use and youth culture during the first tide of drugs.

## The Social Hospital Records and the Historiography of Drugs

Some interpretations of the 1960s and 1970s in general are more dominant than others, and the history of drug consumption in the 1960s and 1970s can also be told in many ways. The historian Andrew Hunt describes the sixties research field as “the myriad of layers of sixties history.”^15^ In Finland, the historian Katja-Maria Miettunen has studied representations of the era. According to Miettunen, popsters, radicals, and writers dominate the image of the past by reminiscing about the era as they remember it – certain personal experiences have excluded others as there are strong constructions of the image of the 1960s.^16^ It seems that some aspects of the 1960s are indeed seen as more relevant than others. Recently, the Finnish historian Henrik Meinander commented on the ways his book about the year 1968 was received. In his book, Meinander aimed to demonstrate that even during times of change, most people continued living their lives as before.^17^ Despite his attempt to prove otherwise, attention focuses persistently on the impact of leftist political forces.^18^ Similarly, the interest in different drug consuming habits has varied. For example, in the Canadian context, the historian Greg Marquis has referred to the “fixation on middle-class youth culture”^19^ in his analysis on drug use studies in the 1970s. Some of the representations of Finnish drug consumption are indeed tied to so-called bohemians, such as writers and theater students who expressed their experiences elaborately,^20^ but they represent a small minority. Katriina Kuusi, a contemporary physician of the era who worked with young drug users in another hospital, has noted that it was hard for many young people to describe their experiences with words:

[…] verbalization seems simply impossible. Action speaks for itself and verbal interaction, when it occurs, is concrete. It is known that young people who are unwell, especially the so-called asocial ones, have poorly developed cognitive and abstract thinking in comparison to their peers. They have difficulties in expressing themselves and difficulties in understanding what their caretakers are saying.^21^

The peak in drug consumption in Finland occurred during turbulent times, and drugs and their users were discussed fiercely. Experts had a significant role in public discussions as well as in the criminalization process that led to the 1972 Narcotics Act (*Huumausainelaki* 41/1972). The Finnish Government had suggested the decriminalization of use, but voting in the Parliament led to criminalization. At the time, the decision differed from the rest of Europe, and in the Nordic countries, only Norway was in favor of criminalization.^22^ In neighboring Sweden, the interpretations varied based on who had the problem before their eyes, which part of the problem was discussed, and who bore the problem – the drug issue was used as political ammunition.^23^ There are many Finnish contemporary sources, as drug consumption was of interest from different perspectives. Journalists wanted to capture the drug phenomenon, and many headlines amplified what is often described as a “moral panic.”^24^ Simultaneously the media evoked interest toward drugs. (At times, the Social Hospital staff stored clippings of hospital patients in between the files.) The drug police knew young people personally, and some police officers have reminisced about the era in their memoirs.^25^ The reportages and memoirs portray the criminalized side of drug use. There are also contemporary theses and scientific studies available, based largely on statistical data and research literature.^26^

There is also more recent research on the topic. For example, the sociologist Jani Selin has studied psychiatric interpretations of drug use and users in Finland,^27^ but his study is based on publications only, and does not cover inpatient care and user experiences. One challenge is that individuals who could share their experiences have not been willing to do so; the historian Mikko Salasuo has pointed out that the topic of drugs is secretive and illegal, and trustworthy sources are hard to find.^28^ A recent work based on oral history has provided valuable information about drug use in the 1960s and 1970s. *Vanha liitto^29^* [The Old League] is a compilation of articles which are based on contemporary interviews. Many of the interviewees have died since the interviews were conducted. The writers of the book emphasize the importance of sensitivity and the value of the way the interviewees wanted to talk about their past.^30^ Remembering certain things and intentionally or unintentionally forgetting others is an essential element of oral history, but the freedom to choose results in selective memories: less favorable ones are left untold.

Although medical sources are mediated, i.e. written mostly by the hospital staff, emphasizing the issues the staff found significant and focusing mostly on the problems of the patients, they offer detailed descriptions of drug use in the 1960s and 1970s, thus widening the historiography of drugs. Medical records represent what are known as expert perspectives on drug use, but hospital records also mediate the experiences of the patients. The sources are thus more elaborate than user experiences: they offer insights to the interaction between the hospital and its patients, as well as societal influences surrounding both. In other words, the hospital records enable the exploration of drug consumption not merely as a “naturalized category,”^31^ which according to the historian Joan Scott would be the risk if experience was taken as the origin of knowledge, but rather as a mixture of different voices, produced as part and parcel of hospital practices. This does not mean that the value of hospital records as sources should be overestimated. The hospitalized individuals are mostly referred to as “patients,” “users,” or “clients” in the sources. This article uses the term patient to underline the medical context and the distanced expert view. Whereas hospitals can simultaneously function as safe places where individuals find security and freedom to talk about their problems^32^ and shelter from street life, it is unlikely that they were open about all aspects of their lives. This could be a result of either unwillingness or inability.

Medical records, including descriptions of height and weight, infections and injection marks, homelessness, and behavior in the hospital widen the perspective of substance use. All of the files – excluding those sent to other hospitals, marked with yellow sheets in between the case files – still exist, unlike many other old medical files in Finland. The Social Hospital was also among the most significant institutions that treated people with drug-related problems, which is why it is a valuable source.

The Järvenpää Social Hospital was situated in the village of Haarajoki, approximately 45 kilometers from the capital city, Helsinki. Patients came from all over the country and in accordance with the 1961 law on the need for care. The patient records between 1965 and 1975 consist of approximately 7000 case files in total, of which only a small portion include drug use, whereas most individuals sought help for their alcoholism, at times combined with the use of tranquilizers or other drugs that were available in pharmacies, with or without a prescription. The numbers are not in line with the proportion of drug problems in Finland, but rather with the hospital policies and capacity.^33^ For example, in 1972, when drug consumption was just about to reach its peak, the hospital had 125 beds, of which 20 were reserved for women, and 40 for “drug abusers.” Altogether 897 patients were in treatment that year.^34^ For this study, I have systematically skimmed through all the available files, aiming at a balanced general view.

The physician-in-chief, Jorma Tirkkonen, was open to different forms of care which were not tied to any one psychiatric doctrine.^35^ The care was guided by the psychiatrist Tirkkonen but nurses and social workers each had their own patients for whom they were responsible. The treatment options included different alternatives such as first aid, group and individual gatherings, religious gatherings, Alcoholics Anonymous, and work therapy. As the historian Heli Leppälä has shown, social policy in Finland stressed the importance of work and productive activities as sources of social esteem and self-gratification: a social right. A normative change in social welfare occurred in the 1960s and replaced the earlier view of work as a duty of the citizen. The change manifested as a new ideal of citizenship: participation.^36^ The Social Hospital staff paid attention to other health and social questions, such as dental care, acquiring new clothes, eyeglasses, and living arrangements after the stay at the hospital. Some of the patients were severely malnourished and were given vitamins in addition to regular meals, and some had infections, gunshot or necrotic wounds, and “liver disease,” i.e., hepatitis, that needed professional treatment. Some patients were sent to other hospitals in case their physical condition was too bad to be treated in the Social Hospital. The records include descriptions of arrangements after the treatment period. Hospitalization also meant making plans for the future. Some patients continued treatment, for example, at *Nuorisoasemat* (Youth Stations), founded in 1970, which were aimed at young people with substance abuse problems.^37^

The records are a mix of information written by the patients themselves, summaries written down by the physician-in-chief, Tirkkonen, and analyses written by nurses, psychologists, and social workers. Many notes were written down using initials only, such as “MLN” meaning social worker Marja-Leena Nousiainen, or “MA,” standing for nurse Mari Aerila. The standardized forms guided the patients’ answers. They included treatment plans, anamneses, tests charting substance abuse, self-evaluation forms, characterological profiles, and forms concerning the former life events of the patient.

## The “Old” and the “New” Drug Use

The change that occurred in use and users between 1965 and 1975 can be seen more clearly when compared to older forms of use. In the following, I exemplify the old use by offering examples of patients who had either been using drugs for a long period of time or whose use differed from the new use in other ways. Then I introduce the new use and discuss how it was seen to differ. Although the division between the old and the new is a slight simplification, both the patients and the hospital staff referred to these two user segments, which is an implication of change.

The older forms of use can be roughly divided into three substance groups in the hospital records. The first group consisted of individuals who used opiates. The consumption of heroin in Finland had been somewhat high already prior to WWII because heroin was used in a common cough mixture.^38^ After the war, heroin was used as a cheap and efficient medicine for many kinds of aches. Although there was a significant rise in the number of opiate users in the post-war years compared to the pre-war years, there were only 165 cases in the capital city area.^39^ The numbers remained similar until the mid-1960s. According to the physician Lenni Lehtimäki’s study, there were approximately 150 methadone users in the mid-1960s.^40^ In other words, although there was a visible problem, it was not a mass phenomenon.

Changes in the opiate scene were in connection to control politics: when one drug became more strictly controlled, the use of another increased. For example, when heroin and morphine became hard to get, different methadone products, including Algidon, Benzalgin, Dolorex, and Palfium, became more common.^41^ Some used amphetamine derivatives alongside opiate use, which included products such as Ritalin, Stimulan, and Methedrin.^42^ These drugs were secondary in importance in comparison to opiates, and were used particularly when stable sources of opiates could not be found, as was also the case among opiate users elsewhere in Europe, such as in West Germany in the 1950s and onwards.^43^

Patients who were in treatment because of opiate consumption often felt alone and differentiated themselves from those in treatment because of alcohol.^44^ They most certainly did not identify themselves as a homogenous group of intoxicant users, as defined in the 1961 law. They also differentiated themselves from the new generation. Many referred to themselves as “classic narcomaniacs” in comparison to the new users, and hospital staff shared this view. Some had started by trying heroin, for example, as a cure for hangovers, and since it became difficult to get heroin, they had switched to Algidon.^45^ Most of the heroin users did not know at the start what they were experimenting with. They were curious or were offered some to try. Some described the post-war times as demoralizing. Some had spouses and relatives who recommended trying heroin, such as uncles and mothers-in-law. The substance history of opiate users shifted from one substance to another, and most of the patients had a long history of use. The substance history could be, for example, as follows: heroin since 1947, from heroin to morphine in 1947, Algidon until 1968. The aforementioned use history had led to fourteen hospitalizations over the course of time, and after every time, the person had started using again. It is likely that frequent hospitalizations influenced the identity of a classical narcomaniac; the term narcomaniac was already a marker of medical influence. Internationally, it referred to opiate use and serious dependency, and in the Social Hospital, classical users had a long history of opiate use in comparison to other drug use.^46^

Johannes,^47^ born in 1921 and an example of an old user, came to the hospital in 1969 together with his wife. He was described as a former alcoholic. For almost twenty years he had also used other substances. He consumed mostly Algidon, but also some stimulants. His social status had suffered. Johannes was described having a “desolated soul” due to drug abuse, and he had a wound in his shin that needed treatment.^48^ According to a memoir by a former drug police officer, such wounds were typical and were not related to syringe-caused infections but had to do with Algidon itself, which apparently had an impact on blood circulation.^49^ In 1969, the hospital notes described both the difficult life of opiate addiction and the old-school attitude of Johannes and his spouse. The excerpt underlines the cultural difference that defined the classical use together with the substance and indicated that the staff shared the idea of a divide between user groups:

The past times have been really difficult [for Johannes] because it has been very challenging to get efficient medicine because Algidon has disappeared even from the “black” [market]. They use any sort of concoctions of different kinds of pills, mixed. They have used Methedrin whenever it has been available. They are not fond of “fashion drugs”, although they have experimented with hashish, marijuana, etc. Amphetamine would be available, but it does not seem to please them either. […] Nowadays it is very difficult to be a narcomaniac in the classical sense.^50^

The second substance user group consisted of patients whose main problem was alcohol, but who medicated themselves with different kinds of pills alongside alcohol. Among these pills were bromide compounds, such as Pentobromital, and meprobamates, such as Nervonus, but the total number of different kinds of pharmacy drugs is vast. The records describe the problems of these patients, for example, as ambivalent emotions. In 1965, a nurse in the hospital wrote notes about a conversation with a patient who thought he was civilized and smart and could not stand people who committed crimes and had to go to prison. (He had committed crimes under the influence, though.) His nurse asked the patient if this feeling was due to scorn toward his own characteristics. The nurse characterized the patient’s problem as a contradiction between “the society party upstairs and the howling wolves in the cellar.”^51^ Contradictions tore many of the patients. More often than not, they had jobs and families, and at the same time, battled with problems in their lives and with their consumption, such as in the following example:

[The patient] Never shows his emotions. He cannot stand his own anger. He might shake because of anger if his wife or daughter disagrees with him. He takes pills and only when he calms down, he says what upset him when he was angry. He is vigorous, resilient, and patient, and likes his job. He thinks he is inferior to others. It is hard for him to get to know other people. […] Nowadays he is nervous, insecure, and shaking. Anxious, self-centered, cannot take any distractions, immediately takes medicine.^52^

The second group self-medicated to a point that medication became a problem in itself. The use took place in solitude and was mostly a means to handle everyday life and its problems.

The third group consisted of individuals who had a habit of sniffing, that is inhaling the vapors of solvents. The division between the third group and the new users is somewhat artificial because the habit sometimes coexisted with other forms of drug use, and some of the patients with a sniffing problem were young, like most of the users in the new scene. Solvents were, however, available even without connections to the drug scene, which is why sniffing was a habit in the countryside, too. In contrast, the new scene was an urban phenomenon.

Some solvent experimentation led to serious brain damage. For example, Kalevi was a 22-year-old sniffer who considered himself to be a hopeless case – he said he would always have access to solvents unless he was in the hospital or prison. Kalevi was also described as an alcoholic who was weak-willed and unbalanced. Sniffing solvent was reported to have had an influence on his learning capacity, and this had resulted in memory loss. After two weeks in the Social Hospital, he caved in and left, stating that he would never get rid of his problem.^53^ More often than not, sniffers were described as shortsighted, and they rarely identified themselves with any ideologies, unlike some of the new users. Inhaling vapors of solvents was thus not portrayed as an essential element of identity, unlike the new drug use.

To conclude, the first group primarily consisted of opiate users who perceived themselves as classical narcomanics. For the second group, consisting of individuals who consumed pharmaceutical medicines, the need for treatment was an embarrassing result of self-medication, which contrasted with the recreational aspects of the new use. These patients lacked the curious, experimental spirit which was typical to the new users, even if some of the abused pharmaceutical medicines were the same. The drug consumption patterns of the young drug users were thus different from the medicine misusers. As Paul Manning has characterized, the social and cultural practices associated with drug consumption lent meaning to the effects of drugs.^54^ The third group inhaled the vapors of solvents and were described as sniffers.

The new use is best characterized as multiple substance use, and perhaps the heterogenic range of substances and the problems they caused inhibited further classifications of user groups in the hospital. The new users encountered classical narcomaniacs, but they did not identify themselves with that group. Instead, the relationship was based on buying and selling of drugs. The sociologist Pekka Hakkarainen emphasizes that cannabis was the flag-bearer in the 1960s drug scene.^55^ Hashish appeared in the hospital records for the first time in 1967, and also lysergic acid diethylamide (LSD) became common over the course of time. However, among the Social Hospital patients, these substances were mostly listed among others. There was no strict differentiation between different kinds of substance use. LSD could be used in addition to hair lotion and ethanol solution. Hashish use occurred together with opiates. Drug use in general was more dependent on availability changes, such as that of amphetamine, and later morphine base, than any particular user identity. Although Hakkarainen emphasizes the role of cannabis during the era, he has paid attention to the problem of representation, as many individuals were out of reach, not included in the statistical data because they did not partake in any societal activities where data for statistics were collected.^56^ The Social Hospital patient records captured descriptions of some of these individuals.

Leila’s case exemplified the new drug use era. In 1969, the nineteen-year-old had been using tranquilizers and stimulants, most of all Valural, which was intended for insomnia. Her list of prescription medicines was long: Diapam, Adinol, Largactil, Melleril, and caffeine. She was dating a drug dealer, who was reportedly also an alcoholic. Her father had been an alcoholic and her parents had separated. According to Leila’s story, her father had been brutal and her mother did not love her. As a child she had been timid and fearful. The teachers had hated her, and her minister did not want her to go through confirmation. During her hospitalization, her character was described as impulsive, easily irritated, and even violent. Her life was not restricted to innocent experimentation: Leila told that since she was fourteen years old, she had been a decoy in a gang. The gang had beaten their victims unconscious and taken their money. A social worker of the Järvenpää Social Hospital analyzed her life as follows:

The thought of healing and the feeling about it has fluctuated. In the gang, she was liked and accepted as she was, and they wait for her to come back with open arms. Elsewhere she is an “enfant terrible.” She is so anxious and sensitive that the only way for her to cope is to act tough, which causes her immense suffering.^57^

Leila’s example shows the complications of the comparison to experimental use. Although Leila might have had long hair and an antiauthoritarian attitude, reflecting the international 1960s fashion, her life was far from expanding consciousness and symbolic use. Leila’s case also introduces us to gangs. Their importance will be analyzed with more detail later. The gangs (*jengit*) were frequently mentioned in the records and had their own role in interpretations. Some were hippie gangs, some just gangs, such as those in the 1970s Helsinki suburbs.

The record of a young man called Matti offers another example of drug use that went beyond experimental. Matti was born in 1948 and had an alcohol problem, but his drug use was the main reason he ended up in the Social Hospital. Matti had been using tranquilizers, stimulants, and hashish since 1968. Notes from another hospital, described in the Social Hospital records, revealed that he had been using Diapam, Triptyl, and heroin, which began to reappear in the global market by 1973,^58^ and amphetamine intravenously. The use had led to various suicide attempts, arrests, physical injuries, and mental problems. He was forced to come to treatment, as it was believed he would not come voluntarily.^59^ Leila’s and Matti’s cases show that hospitalized individuals used a variety of substances, used drugs in bigger groups, and used psychedelic drugs for reasons other than their countercultural appeal. The next two sections delve into their lives in more detail by offering personality descriptions that were written down by the hospital staff, as well as the ideological explanations for use offered by both the patients and the staff.

## The Death of Self and Other Personality Interpretations

The Social Hospital records avoided categorizing the patients into any specific user types based on personality.^60^ Although diagnoses were marked in each file, most of the content of the files was descriptive, focusing on social issues instead of psychologizing behavior, and paying attention to the ways the patient characterized his or her life. This was in line with the more general attitude in the social work scene, as it underlined the social character of the “epidemy” and avoided oversimplified labels.^61^ Similarly, the 1960s and 1970s witnessed other significant changes. For example, by the latter half of the 1960s, the discussion related to the concept of deviance decreased the emphasis on pathologizing and included a wider range of interpretations.^62^ Despite the similarities with the more general trends, the Social Hospital records offer a significantly different perspective to other psychiatric interpretations of the era. According to Jani Selin, the significance of childhood and the family were emphasized in psychiatric writings on drug addiction.^63^ In the Social Hospital records, family played only a minor role, and interpretations of the patients were not explicitly psychodynamic. Eclecticism characterized the patient descriptions.

Throughout the twentieth century, Finnish mental hospitals had admitted patients who suffered from their drug consumption. Among typical diagnoses was that of psychopathy, which was used to explain causes behind substance use.^64^ In the Finnish version of the International Classification of Diseases (ICD-6), adopted in 1954, substance abuse was covered with one diagnosis (numbered 323) titled *Abusus alius venenorum*, *medicamentorum*. It included the following specifications, which gives a sense of the substance spectrum: *Barbiturismus*, *Bromismus chronicus*, *Cocainismus*, *Codeinismus*, *Dolantinismus*, *Heroinismus*, *Morphinismus*, *Nicotinismus*, *Opiismus*, and *Phenedrinismus*. An “etc.” was added in the end to indicate that there were more abused substances in the field.^65^ In ICD-8, adopted in 1969, the former category of medicine and “poison” abuse was replaced by *Narcomania, abusus medicamentorum* (numbered 304), and it had nine subcategories, now including also hallucinogens and cannabis.^66^

The diagnosis of psychopathy was abandoned when ICD-8 was adopted.^67^ In the Social Hospital, the most common diagnosed form of psychopathy, until the classification change, was 320.7, which was specified as restlessness. Character neuroses (numbered 318) were also common. From 1969 onwards, the diagnoses had different names, but in practice, the new “pathological personalities” in ICD-8, referred to with the number 301, were much like the former subcategories of psychopathy. Among common pathological personality descriptions in the Social Hospital sources were asthenic, immature, affective, schizoid, explosive, and paranoid types. Besides pathological personalities, different types of neuroses, referred to with the number 300, were diagnosed, including phobic, obsessive-compulsive, neurotic depressive, hypochondria, and *angoris*, which referred to anxiety.

Despite using diagnoses, commonly divided into three diagnostic groups to specify the psychiatric, somatic, and abuse-related problems, the psychiatric evaluations of patients were short. However, the descriptive style of the Social Hospital records does not mean there were no interpretations of the patients’ personalities and the influence the drugs imposed on them. According to some descriptions, drugs had changed the personality of the patient. According to others, the patient possessed characteristics that led to drug use. Some cases were not connected to personality at all. The influence of gang or other “environmental factors,” such as experiences in reform schools and mere curiosity and willingness to experiment with drugs, were common explanations for use. In some cases, the staff paid attention to young age and immaturity as either temporary or permanent characteristics. In others, the hospital staff suspected that the patient had incipient schizophrenia, and some personality changes were due to brain damage, caused by sniffing solvents among other use, or some physical injury. These commonly cited causes are now introduced in more detail.

The personality descriptions included frequent characterizations such as infantility, difficulties expressing oneself, sensitivity, volatility, lack of love toward oneself, inner conflicts, irritability, unrealism, and insecurity. These were characteristics that were seen to influence the drug problem. Dependency was also commonly interpreted. In some cases, the hospital staff hoped that the patients would learn to take responsibility for their own actions. Some, according to their experience, had the risk of becoming institutionalized and leaving the responsibility to others. One patient had stated that it was safe and worry-free to stay in the hospital, which was interpreted as another form of dependence, typical to the patient.^68^ Dependence was one of the common terms in medical language in Finland at the time; drug use was explained as either passive-dependent or passive-aggressive.^69^ Similarly, the hospital records described patients as dependent on others and on their acceptance. They felt unsafe, insecure, and unattached on their own, as if they did not belong anywhere. This tendency was described in wider spheres, too: the journalist Sirkka Germain, who interviewed “drug youth” in the 1970s, characterized dependence on institutions as “institution addiction” which could replace other forms of addiction.^70^ In other words, institutions created a new kind of dependency.

Gangs were seen as the most typical form of dependency. One’s position in the gang could be contradictory in many ways. Patients often believed that the other gang members would rejoice if they stayed clean. Others were in doubt and even afraid of the gang. They could also have differing views about themselves, such as Raija, who wanted to be tough and a “good girl” at the same time. She was described as lacking other people’s friendliness and appreciation: “weak self-image, strong sense of inferiority, possibly for this reason she got into asocial company.”^71^ Wanting to belong to a gang could also be interpreted as indicating a lack of lasting relationships. In many cases, the hospital staff emphasized that patients should take responsibility for their own lives, despite bad childhood experiences, although they did pay attention to the family, such as in the following example:

Apparently the upbringing in the family has been inconsistent and the client has not been supported enough at the critical stages of childhood and puberty, which had led to seeking out the gang and to susceptibility to the “drug contagion”. […] The client has striking difficulties to talk about his problems, at least to older people, whereas he gets along better with people of the same age. He tries to become independent, but has dependency problems in relationships.^72^

This example also shows that the patient’s family background was acknowledged, but the focus was on the here and now.

Teppo’s use history was explained in 1975 by his need to belong, which had led to keeping the wrong kind of company in a gang. This had had many consequences, which were listed in his record: “the death of self and own personality, roles, fear, schizophrenia, hallucinations, passivation, hepatitis, losing weight, poor fluid balance, impaired capacity, worsening relations with the family.” Teppo had operated in a “drug dealing organization” and had received a criminal sentence. He was not sure whether he wanted to change his lifestyle or not. The hospital paid attention to his different roles: “The client is a sharp, sympathetic, and pleasant young man who is aware of his charm and can make use of it, even in wrong ways, when needed.”^73^

Another patient, hospitalized in 1971, was waiting to be sentenced. The staff hoped that staying clean in the prison would help the patient to think about his life. Such optimism was often pointless as it became evident that some patients learned to use new substances while in prison. There were, however, those who stayed clean and found new perspectives to their lives. The patient’s passiveness was emphasized by a nurse, referring to the idea of passive dependency: “It feels like he was a small helpless boy who acknowledged his problems to an extent but lacked belief in his own opportunities. The drug user world is too captivating at this point. Passive and suspicious. He can’t stop using on his own, and he does not have the patience to focus on treatment yet.”^74^

Some of the personality characteristics were explicitly explained through drug use. The characterizations were commonly in connection to opiate and amphetamine use. These characteristics included timidity, poor emotional life, shortsightedness, insecurity, expressionlessness or frozenness, volatility, restlessness, inability to listen, and a tendency toward depression. Some seemed to want to control others – as Teppo had also done. One patient, for example, was described to have the ability to be “tough and ruthless.”^75^ Ruthlessness could also manifest as charming and manipulating the staff, and even explicitly stated narcissism.^76^ It seems that during this era, the hospital staff increasingly became a target of manipulation, which implies that manipulative behavior in the hospital evolved into a feature of drug use identity.

As the Social Hospital was not a mental hospital, it did not focus on serious forms of mental illness. Symptoms of mental illness were, however, common. Kari, hospitalized in 1973, was suspected of having incipient schizophrenia. He had spent time in Sweden, where he used morphine base, a brown, non-water-soluble powder that had arrived in the drug market by the 1970s. Kari was very skinny, and when he got hepatitis, he came back to Finland and stopped using amphetamine. Now he was mostly using hashish. Kari said his liver was “broken” and doctors would not believe him. This is why he had his own special diet to avoid foods that were dangerous to his liver, as is described in the following excerpt, written by a social worker: “He speaks a lot about his illness and how ill he is and how carefully he has to choose his food, he also speaks about occultism, clairvoyance, and how he is not afraid of death. Life goes on, there is only some physical limit that one crosses when moving on to another life.” ^77^ The hospital staff believed that Kari understood realities and understood where he was, but reality and unreality were mixed in his stories. He also did not join other patients. Instead, he spent most of the time in his room. There were also patients who evidently hallucinated. One of the patients, unlike Kari, joined group meetings and seemed to be in his own world. The patient always answered questions, but his answers had nothing to do with them. The hallucinations appeared pleasant.^78^ In such cases, it was far from simple to differentiate psychotic symptoms from the influence of substances. Hospitalization was an opportunity to monitor if somatic care and the lack of drugs changed symptoms, but more often than not, the patients smuggled, or were suspected of smuggling, substances into the hospital and either got caught and were made to leave the hospital, or left prematurely.

One strand in drug-related public debates in the 1960s and 1970s was the “elitism” connected to middle-class use. Elitism was a commonly used term at the time, a weapon in debates between the experts. The elite consisted of generational protest against society, experimenting youngsters, who were considered to be above bigger problems, the “crisis group.”^79^ Some of the patients indeed seemed to be above their problem and did not view it as society did. However, it was rare that the patients thought they were in a position to criticize the hospital from an elitist perspective, and the hospital did not take part in this strand of drug identity discussions. One such rare case was Santeri, hospitalized in 1969, who reported his opinions about the hospital and his own condition:

“The food is good and most of the staff is friendly, but the intelligence of some of the male caretakers should be higher. This should be taken into account when choosing the staff and in their training.” The patient has not had any problems with the staff, but he looks at the issue from the institution’s perspective. Regarding his abuse, he says that he consumes a bottle of vodka in a month as an appetizer and takes stimulants to be effective.^80^

Santeri even diagnosed himself as a neurotic and confirmed that he was not psychotic. He thought that Ritalin and Methedrin were the best drugs for him. He could even synthetize Methedrin and explained how temperature had a crucial significance in the process. He listed different kinds of drugs and their ingredients. The report about Santeri, written by a nurse, ended: “He laughs and reckons I am not as clever as him. I agree.”^81^

Despite Santeri’s superior attitude, he was not able to control his substance abuse. It is also important to note that with better safety nets, some elitists had other ways to deal with their substance abuse problems, and they did not necessarily end up in hospitals. In fact, some even worked in hospitals as physicians.^82^ Many patients were described as having potential and even supportive family backgrounds, but the severity of the drug problem dominated the prognosis. For example, a young woman was described by a social worker as follows: “intelligent, verbally talented, artistic, manipulative young lady whose own emotions are somewhere deep under the surface.”^83^ By the time drug users were hospitalized, they had already fallen out of safety nets and regular life including work or studying. Instead, the patients seemed to have their own worldview in which drugs had a focal role.

## Drug Philosophy

In the United States, the promoters of LSD in the early 1960s believed in the transformation of American society.^84^ To the post-war generation in Germany, drug consumption was about opting out of consumer society, although their actions created a global market in recreational pharmaceuticals.^85^ “Exi” was a term that was used to describe individuals who were young, educated, middle class, and into French existentialism and jazz.^86^ The scene in the Netherlands was divided into “psychedelics,” who used LSD and marijuana, and people from lower social classes, who used amphetamines or opium.^87^ There are glimpses of the ideological backgrounds for drug use among the hospitalized individuals, described either by the Social Hospital staff or the patients themselves, but they can be summarized as shallow. For many, the explanations had a recreational background: using drugs was a way to get rid of “gray everyday life.”^88^ Some explained that marijuana felt soothing and helped in falling asleep, and it was not any danger to others, unlike alcohol, which could cause harm to other people. Others related that they were able to enjoy movies and experienced “great feelings” when they were high.^89^ Drugs were also related to the expansion of self-knowledge, and they helped users to be in other people’s company without anxiety.^90^

According to Mikko Salasuo, the hippie movement and underground scenes never became strongly rooted in Finland. The ideology and psychedelia that were connected to drugs were in the background, whereas mere curiosity explained most drug experimentation. The cultural atmosphere was to a great extent negative toward drugs. Salasuo explains that the student movement and the influence of the Finnish Union of Secondary Students inhibited interest in the drug scene.^91^ On the other hand, Pekka Hakkarainen has emphasized how liberalism, hippie ideology, and an underground spirit fueled interest in experimenting, which then led to further experimentation in most cases, and more regular use in others.^92^ The influence of the international underground phenomenon on drug consumption thus depends on the perspective.

It is evident already from the patient record descriptions that the international hippie movement, or at least the fashion, inspired young Finns. The patients were described wearing red silk shirts, see-through dresses, short skirts, and keeping their hair long. Many lived in communes, slept in train carriages with different people, and more generally associated with hippies. The described looks of the patients carry whiffs of the underground-inspired era. The staff often referred to either “drug philosophy” or “drug ideology” in the records, such as in the following example of a social worker’s report: “Drug use has led to trouble with the authorities, but otherwise [the patient] does not seem to have suffered from use. On the contrary, he seems very attached to drugs and the drug world, its ideology.”^93^ The term drug philosophy was likewise used without further explanation, such as in the following, written down by the same social worker: “Clearly fond of drug philosophy and leans on it – getting rid of drugs is hard.”^94^ Although the terms drug philosophy and drug ideology were never explained explicitly, they clearly expressed something to other staff members: there was consensus in the hospital and perhaps also wider in the community, about what the terms entailed. The medical publications in the 1960s and 1970s in Finland referred to “drug epidemy,” which exposed Finnish youth to the kinds of fashions that were previously unfamiliar in the country. The medical writings also included long lists of societal ills, such as fear and alienation, insecurity, superficial contemporary culture, the media, rapid structural changes, and economic profit gain.^95^ The views reflected media interpretations both nationally and internationally.

The records mediate some impressions indirectly. Some patients were either hostile toward the current state of affairs in society, or pessimistic about their own role as part of it. Their thoughts resembled the so-called dropout ideology: turn on, tune in, drop out, as the icon of psychedelia Timothy Leary stated. This could be in many cases simplified as unwillingness to work. The patients characterized their use as escape from reality or rebellion against society. One of the patients explained: “I have always been bad at working and all of my friends are junkies.”^96^ He was described having an ideology that considered everyone as good and lovable. According to a social worker, there were repression mechanisms in his thinking. As a child he had escaped to a fantasy world and was now “living it” – the drugs served as the mechanism.^97^

At the turn of the decade, the young man Heikki dreamed of a different kind of society. Most of all, he thought, different kinds of substances – alcohol, hashish, and marijuana – should be free so that he would not have to be afraid to use them. Although Heikki had, according to his hospital records, suffered from “stubborn hallucinations,” he did not admit that drugs could be dangerous to his health. At least according to the descriptions by a psychiatric nurse, it seems that Heikki’s antagonistic relationship with society was mostly tied to drug use, which is evident in the following description:

What use is it to get rid of drugs and come back to this sick society. [The patient] Says that all of those who do not allow the use of hashish but punish the users support the sick society. Hashish use should be allowed. [The patient] Says that he is going to keep on using substances. Is not interested in treatment options because thinks that everyone who is over 20 years old is already corrupted by the sick society, and for this reason there are no healthy employees who could help him.^98^

It is not evident from the source whether “sick society” was an educated reference to Erich Fromm and his ideas about alienated and inadequate sick society, or if Heikki had picked up the expression from the streets.^99^ It is, however, likely that the expression originated from Fromm and his book, *The Sane Society*, published in 1955, which was also translated into Finnish in 1971. There were similar statements that seemed to be connected to the hippie lifestyle. One patient summed up his contempt toward society and its “mental disease” that manifested as the over-appreciation of productive work and was targeted at the patient’s psyche.^100^ Referring to one’s psyche in the Social Hospital was a kind of play with psychiatry. The physician-in-chief Tirkkonen described another patient who wanted to live with the hippies, who the patient admired. According to the patient it was easy to live with them and they were financially independent. “This was a kind of way to object to society,” Tirkkonen summed up.^101^ One patient who said he could not stand the prevailing system chose drugs and “apparently eastern philosophy,” his record stated. He had worked once in his lifetime for a month, since then he had opposed working, including work offered as a form of therapy in the Social Hospital. According to the patient, it was wrong to bring up the work issue, because he had his prison experiences and forced labor in mind.^102^ To the patient, the Social Hospital was just another form of societal oppression. The Finnish forms of societal critique seemed to have been in line with international trends, with some national flavor. In comparison, in Spain, drug consumption was a means of identity construction during the Spanish transition to democracy in the latter half of the 1970s.^103^ The drug culture took local forms.

Although the hospital staff had their own views about gangs, to the patients gangs represented life that was out of the ordinary and support to those who did not think they were suited for an ordinary life. A girl who had used various kinds of tablets, injections, and alcohol since she was fifteen years old described the gang members as “akin.”^104^ By the mid-1970s there were infamous gangs in suburbs like Maunula, less than 10 kilometers away from the Helsinki city center. In 1975, one of the gang members contemplated his life as follows: “What is the point in trying when you know that you will soon be back in prison.”^105^

Pessimism toward societal expectations was evident as hopelessness regarding work life. One patient described herself as shortsighted and bored; there were no jobs that the patient felt like doing.^106^ Another patient felt that he had wasted his whole life and considered himself “too weak for the hard world.”^107^ Although the majority of the patients were either unwilling or unable to work or study, there were some who had held jobs. One of them described how he had pressure at work because he could not keep up with technology, “run after automated machines,”^108^ referring to modernization.

At least according to the hospital notes, the antisocial attitude of some patients derived from becoming estranged from their parents. Rather than interpreting psychodynamic family relations, the records, written by a social worker, describe the rebellion of the younger generation. Sirkku, hospitalized in 1972, had problems accepting her parents’ “materialistic lifestyle” from the time she was fourteen years old. Sirkku’s father was an active member of the conservative National Coalition Party; she had joined the socialist Finnish People’s Democratic League.^109^ She also left the national church. Sirkku explained that when she was younger, she did things just to be in opposition, but she believed that later she had internalized her (political and other) values. While Sirkku was hospitalized, she got engaged to a “reform school boy” after knowing him for only two weeks.^110^ The hospital notes suggest that she did not recognize that her decision to get engaged had anything to do with her oppositional attitude.

At times, the rebellious attitude was also targeted at hospital staff. One of the nurses described her interaction with a patient who did not either remember or want to come to his appointments. When the patient came to the room, he sat down rakishly, lifted his smelly feet on the table and asked, scornfully smiling, what she wanted from him:

The patient asks in a challenging tone if I am trying to adjust him to society. I talk about learning the least amount necessary to live in contemporary society, such as getting out of bed in the morning. The patient tells me he will be okay and will never get up early in the morning. Then he tells me about his activities in a [political] party and his daily routines as part of it. I say that if this is the way it is, then getting up early is not needed. This is how I comply with all of the patient’s “socializing attempts.” When the patient tells me that he isn’t interested in anything, I say there is nothing that can be done, then. When the patient got permission to sleep later in the morning, he wanted the alarm set for 7:15 am. Based on all this, he seems to have a need to behave opposite to what is expected of him.^111^

Unlike manipulation, which indicated that for the patients, the hospital was just another entity that could be tricked and fooled, rebellion was a more emotional form of behavior and indicated a personal relationship with the hospital and its staff members. As the excerpt shows, at times the patients were listening, even if they wanted to show they were doing the contrary.

## Conclusion

The 1960s and 1970s was an era that witnessed an unprecedented rise in drug consumption. It was also a time when new kinds of drug user identities were only beginning to take shape. The hospital staff and the patients shared knowledge about the drug consumption phenomenon. The hospital workers were perhaps even the only listeners of young people living in the streets. Standardized forms helped to document drug user experiences that were otherwise difficult to obtain.

The main argument of this article has been that the Social Hospital archive offers an invaluable and versatile source to the lives of young drug users in the 1960s and 1970s. The medical point of view emphasized both the pathological personality traits on one hand and the influence of drugs on personality on the other. The lifestyle-based understanding of the drug phenomenon focused on avoidance of work and the unwillingness to adjust in “sick society.” Also gangs played an important part in drug use. Unlike in the heated debates that took place in the public, the Social Hospital portrayed drug use as a many-sided phenomenon. The Social Hospital archive does not align with the experimental narrative of drug use either, for which the 1960s and 1970s are better known. Most of the patients had mental, social, and somatic problems, which were seen to require hospitalization. Hospitalization was an indication of a process that was heading toward marginalization.

By the latter half of the 1970s, drugs went out of fashion. In Finland, even cannabis consumption reduced radically. According to the historian Mikko Salasuo, this was because alcohol consumption became more common, the original ideology connected to drug consumption vanished, some users moved on with their lives and got jobs instead of remaining in the counterculture spheres, use was criminalized and more strictly controlled, there was negative publicity on one hand and active prevention campaigning on the other, and youth culture became commercialized, offering new trend alternatives.^112^ Or, as Robert Stephens has graphically put it, the image of students smoking joints and expanding their consciousness changed into working-class youths sticking needles into their arms, turning to crime, and dying in the streets.^113^ Drugs no longer appealed to the masses. For some years after 1975, drug consumption in Finland turned back into a marginal problem that only affected outcasts. The 1972 Narcotics Act, however, remained significant, as drug use is still criminalized, which has affected the next tides of drugs that followed. One can only ask whether the vote for the criminalization of use would have been any different had the drug users been heard. Maybe now would be a good time to ask, listen, and rethink the grounds for the criminalization of drug use that at the time were based mostly on opinions, not knowledge.

## Footnotes

1 Pia Rosenqvist and Kerstin Stenius, “Medicalisation of the Social Perspective: Changing Conceptualisations of Drug Problems in Finnish Social Care and Substance Abuse Treatment,” *Nordic Studies on Alcohol and Drugs* 31 (2014): 551–568.

2 Robert P. Stephens, *Germans on Drugs: The Complications of Modernization in Hamburg* (Ann Arbor: University of Michigan Press, 2007), 13.

3 On drugs in Finland until 1950, see Mikko Ylikangas, *Unileipää, kuolonvettä, spiidiä: Huumeet Suomessa 1800–1950* (Jyväskylä: Atena, 2009).

4 Alexandre Marchant, “When ’Drugs’ Become ’Drugs’: Issues of Pharmaceutical Abuse in France from the 1960s to the 1990s,” *Medicina nei Secoli arte e scienza* 26 (2014): 557–596.

5 David Herzberg, “Boundaries in the History of Alcohol, Drugs, and Medicines,” *Social History of Alcohol and Drugs* 26 (2012): 117–121.

6 Johan Edman and Kerstin Stenius, “Conceptual Carpentry as Problem Handling: The Case of Drugs and Coercive Treatment in Social Democratic Welfare Regimes,” *International Journal of Drug Policy* 25 (2014): 320–328.

7 Pekka Hakkarainen, *Suomalainen huumekysymys: Huumausaineiden yhteiskunnallinen paikka Suomessa toisen maailmansodan jälkeen* (Helsinki: Alkoholitutkimussäätiö, 1992).

8 Liite no 1. Päihdetoimikunnan asettama valmisteleva työryhmä 2, luonnos yhteenvedoksi. Huumausaineneuvottelukunnan arkisto. Neuvottelukunnan asiakirjat. The National Archives, Helsinki.

9 The Järvenpää Social Hospital Archives will henceforth be referred to with the abbreviation JSHA.

10 The hospital is still operational and known now as Addiction Hospital, run by A-Clinic Ltd.

11 Ilkka Halonen, ”Hoitotyön perinteestä Järvenpään sosiaalisairaalassa,” *Järvenpään sosiaalisairaala 30 vuotta* (Järvenpää: Järvenpään sosiaalisairaala, 1981), 11–20.

12 The research permit (THL/1752/5.05.00/2018) was granted by the Finnish National Institute for Health and Welfare. The following laws were taken into notice: (2016/679), (785/1992), (812/2000), and (523/1999). See https://finlex.fi/en/.

13 Mikko Salasuo, ”Tajunnan kumous: Ensimmäinen huumeaalto,” in *Arkinen kumous: Suomalaisen 60-luvun toinen kuva*, ed. Markku Peltonen, Vesa Kurkela, and Visa Heinonen (Jyväskylä: SKS, 2003), 84–109; Hakkarainen, *Suomalainen huumekysymys*, 58–63.

14 Paul Manning, "An Introduction to the Theoretical Approaches and Research Traditions," in *Drugs and Popular Culture: Drugs, Media and Identity in Contemporary Society*, ed. Paul Manning (Devon: Routledge, 2007), 3–28.

15 Andrew Hunt, "‘When Did the Sixties Happen?’ Searching for New Directions," *Journal of Social History* 33 (1999): 147–161. On researching the sixties, see also Nancy Janovicek, “’If you’d told me you wanted to talk about the ‘60s, I wouldn’t have called you back’: Reflections on Collective Memory and the Practice of Oral History,” in *Oral History Off the Record: Toward and Ethnography of Practice*, ed. Anna Sheftel and Stacey Zembrzycki (New York: Palgrave, 2013), 185–200.

16 Katja-Maria Miettunen, *Menneisyys ja historiakuva: Suomalainen kuusikymmentäluku muistelijoiden rakentamana ajanjaksona* (Helsinki: SKS, 2009).

17 On the topic, see also Henrik Meinander, *Samaan aikaan: Suomi ja maailma 1968* (Helsinki: Siltala, 2019).

18 Henrik Meinander, ”Torjuntavoitosta Vanhan valtaukseen,” *Historiallinen Aikakauskirja* 118 (2020): 237.

19 Greg Marquis, “Constructing an Urban Drug Ecology in 1970s Canada,” *Urban History Review* 42 (2014): 27–40.

20 There is even a study in which theater students and their drug consumption habits are compared. Pekka Kopteff, *Opiskelijoiden lääkeaineiden ja huumeiden väärinkäyttö* (Tampere: Sosiaalipolitiikan laitoksen tutkimuksia, 1976). See also Marcus Boon, *The Road of Excess: A History of Writers on Drugs* (Cambridge, MA: Harvard University Press, 2002).

21 Katriina Kuusi, ”Nuoret hoidossa,” in *Psykoterapeuttinen aikakauskirja 2* (Pieksämäki: Therapeia-säätiö, 1972).

22 See e.g., Hanna Putkonen and Katariina Parhi, ”Lääkärit ja puoli vuosisataa suomalaista huumausainepolitiikkaa,” *Yhteiskuntapolitiikka* 84 (2019): 635–639; Pekka Hakkarainen, ”Hoito vai rangaistus? Käytön kriminalisointikeskustelu vuoden 1972 huumausainelain eduskuntakäsittelyssä,” in *Muutoksen pysyvyys: Sosiologisia näkökulmia yhteiskuntaan*, ed. Osmo Kivinen (Turku: Turun yliopiston, 1990), 277–301; Hakkarainen, *Suomalainen huumekysymys*.

23 Johan Edman and Börje Olsson, “The Swedish Drug Problem: Conceptual Understanding and Problem Handling, 1839–2011,” *Nordic Studies on Alcohol and Drugs* 31 (2014): 522–523; Johan Edman, “An Ambiguous Monolith: The Swedish Drug Issue as a Political Battleground, 1965–1981,” *International Journal of Drug Policy* 24 (2013): 464–470.

24 On ”moral panic,” see Salasuo, “Tajunnan kumous,” 105; Andrew Blake, “Mental Health and Moral Panic: Drug Discourses in History,” in *Drugs and Popular Culture*, 29–45; Jock Young, “Moral Panics and the Transgressive Other,” *Crime, Media, Culture: An International Journal* 7 (2011): 245–258. The contemporary newspapers, magazines, and tabloids are full of examples where drugs are portrayed as a scandal. There is also a book dedicated to drug life, written by a journalist. See Sirkka Germain, *Huumeruletti pyörii* (Helsinki: Tammi, 1980).

25 For example, Risto Nordman and Harri Nykänen, *Huumekyttä: Rikospoliisin muistelmia* (Juva: WSOY, 1991); Torsti Koskinen, *Helsingin huumeet 1966–1996* (Helsinki: Sisäasiainministeriö, 1997).

26 See e.g., Ritva Uusitalo, *Nuoret ja huumausainelaki: Huumausainelakia koskevien tietojen välittyminen osana nuorten sosiaalistumista* (Helsinki: Helsingin yliopisto, 1981); Kerttu Louhivuori, *Drug-Addicts and Non-Addicts among Finnish Pubertal Adolescents: A Comparative Sample Study* (Helsinki: Helsingin yliopistollisen keskussairaalan psykiatrian klinikka, 1971); Tor-Björn Hägglund and Kari Pylkkänen, *Nuoret ja huumeet: Nuoriso-ongelmien psykososiaalinen tausta ja ennaltaehkäisy* (Porvoo: WSOY, 1976); Pirkko and Juhana Idänpään-Heikkilä, *Huumeet: Asiaa huumausainekysymyksestä* (Porvoo: WSOY, 1974); Paula Määttä, *Images of a Young Drug User* (Jyväskylä: University of Jyväskylä, 1976); Ilkka Taipale, *Huumausainekysymys* (Porvoo: WSOY, 1970). For a comprehensive list of publications, see Mikael Stenius, ed., *Huumebibliografia 1850–1989* (Jyväskylä: Kellokosken sairaala, 1990).

27 Jani Selin, ”Lääketiede, huumeriippuvuus ja huumeriippuvuuden hoito Suomessa 1965–2005,” *Sosiaalilääketieteellinen Aikakauslehti* 47 (2010): 250-265.

28 Salasuo, ”Tajunnan kumous.”

29 Heini Kainulainen, Jenni Savonen, and Sanna Rönkä, ed., *Vanha liitto: Kovien huumeiden käyttäjät 1960–1970-lukujen Helsingistä* (Helsinki: SKS, 2017).

30 Heini Kainulainen, Tuula Kekki, Aarne Kinnunen, Sanna Rönkä, Jenni Savonen, and Jussi Perälä, ”Huumeiden käyttäjät silmätikkuina,” in *Vanha liitto*, 13.

31 Joan W. Scott, ”The Evidence of Experience,” *Critical Inquiry* 17 (1991): 773–797.

32 On hospitals as safe places, see Katariina Parhi, “Boyish Mannerisms and Womanly Coquetry: Patients with the Diagnosis of Transvestitismus in the Helsinki Psychiatric Clinic in Finland, 1954–1968,” *Medical History* 62 (2018): 50–66.

33 Pekka Hakkarainen has made a summary of some contemporary studies regarding the prevalence of drug experimenting. Hakkarainen, *Suomalainen huumekysymys*, 63.

34 Jarmo Kekki and Kirsti Uitto, *Seurantatutkimus vuonna 1972 Järvenpään sosiaalisairaalassa hoidetuista potilaista* (Järvenpää: Järvenpään sosiaalisairaala, 1974), 10–11.

35 Jukka Ahonen, *Järvenpään sosiaalisairaala: Päihdehuoltomme keskeisen laitoksen vaiheita* (Saarijärvi: A-klinikkasäätiö, 1999), 84–89.

36 Heli Leppälä, “Duty to Entitlement: Work and Citizenship in the Finnish Post-War Disability Policy, Early 1940s to 1970,” *Social History of Medicine* 27 (2013): 144–164.

37 On Youth Stations see e.g., Jukka Ahonen, *Päihdehuoltoa rakentamassa: A-klinikkasäätiö 1995–2005* (Jyväskylä: A-klinikkasäätiön raporttisarja nro 51, 2005), 185–194.

38 Achilles Westling and Jaakko Riippa, “Pre-war and Post-war Narcomania in Finland,” *Annales Medicinae Internae Fenniae* 45 (1956): 151–174.

39 See Lenni Lehtimäki, ”Narkomaniasta Suomessa,” *Alkoholikysymys* 34 (1966): 119–128; Westling and Riippa, ”Pre-war and Post-war Narcomania in Finland.”

40 Lenni Lehtimäki, “Narkomaniasta Suomessa.”

41 Hakkarainen, *Suomalainen huumekysymys*, 53–57.

42 JSHA N54/70.

43 For information on West Germany, see Stephens, *Germans on Drugs*, 32.

44 E.g., JSHA 3/65.

45 Ibid.

46 See e.g., Edman and Olsson, “The Swedish Drug Problem,” 508. Prewar use in the Nordic Countries was perceived differently. See Jesper Vaczy Kragh, “Social Class and Opiate Addiction in European Psychiatry, 1870–1955” in *Social Class and Mental Illness in Northern Europe*, ed. Petteri Pietikäinen and Jesper Vaczy Kragh (New York: Routledge, 2019), 115–132.

47 All the patient names in this article have been changed and any personal data that could lead to identifying the cases were removed during archival work.

48 JSHA 58/69.

49 Nordman and Nykänen, *Huumekyttä*, 9.

50 JSHA 58/69.

51 JSHA 364/65.

52 JSHA 458/69.

53 JSHA 8715.

54 Manning, “An Introduction to the Theoretical Approaches and Research Traditions,” 9.

55 Hakkarainen, *Suomalainen huumekysymys*, 68.

56 Ibid., 83.

57 JSHA 19/69.

58 Stephens, *Germans on Drugs*, 110.

59 JSHA file 8634.

60 The same does not apply to analyses of alcohol consumption as the hospital had categorizations for different types of drinking, including “agony drinker” and “lust drinker.”

61 Rosenqvist and Stenius, “Medicalisation of the Social Perspective,” 557, 564.

62 Katariina Parhi and Mikko Myllykangas, “Liberating the Deviants: How to Change the Politics of Social Control – A Case Study from Finland, 1967–1971,” in *Social Class and Mental Illness in Northern Europe*, 194–213.

63 Jani Selin, ”Lääketiede, huumeriippuvuus ja huumeriippuvuuden hoito Suomessa 1965–2005.”

64 Katariina Parhi, *Sopeutumattomat: Psykopatian historia Suomessa* (Helsinki: Siltala, 2019), 54–59; Selin, ”Lääketiede, huumeriippuvuus ja huumeriippuvuuden hoito Suomessa 1965–2005,” 252–254.

65 *Tautinimistö – Nomenklatur av sjukdomar* (Helsinki: Lääkintöhallitus, 1953).

66 *Tauti- ja kuolinsyyluokitus* (Helsinki: Lääkintöhallitus, 1969).

67 Katariina Parhi and Petteri Pietikäinen, “Socialising the Anti-Social: Psychopathy, Psychiatry and Social Engineering in Finland, 1945–1968,” *Social History of Medicine* 30 (2017): 637–660.

68 JSHA 429/72.

69 Selin, ”Lääketiede, huumeriippuvuus ja huumeriippuvuuden hoito Suomessa 1965–2005,” 253.

70 Germain, *Huumeruletti pyörii*, 173–174.

71 JSHA N109/74.

72 JSHA 142/75.

73 JSHA 722/75.

74 JSHA 223/71.

75 E.g., JSHA 404/75; 152/75; 654/74; 173/71.

76 JSHA 108/75; N71/75.

77 JSHA 41/73.

78 JSHA 68/75.

79 See e.g., Rosenqvist and Stenius. “Medicalisation of the Social Perspective,” 557.

80 JSHA 27/69.

81 JSHA 27/69.

82 This is evident through expert interviews, which will be discussed in the future publications.

83 JSHA 96/75.

84 Robert C. Cottrell, *Sex, Drugs, and Rock’n’Roll: The Rise of America’s 1960s Counterculture* (Lanham: Rowman Littlefield, 2015), 76-77.

85 Stephens, *Germans on Drugs*, 6.

86 Ibid., 63.

87 Gemma Blok, “Pampering ‘Needle Freaks’ or Caring for Chronic Addicts? Early Debates on Harm Reduction in Amsterdam, 1972-82,” *Social History of Alcohol and Drugs* 22 (2008): 245.

88 E.g., JSHA 514/75.

89 E.g., JSHA N74/74.

90 JSHA 94/75.

91 Salasuo, ”Tajunnan vallankumous,” 96–99. On the Finnish Union of Secondary Students, see Essi Jouhki, *Teinikuntatoiminnan sukupolvet: Muistitietohistoria oppikoulujen koululaisliikkeestä 1950–1970-luvuilla* (Oulu: Acta Universitatis Ouluensis 2020): http://jultika.oulu.fi/files/isbn9789526225173.pdf.

92 Hakkarainen, *Suomalainen huumekysymys*, 88.

93 JSHA 152/75.

94 JSHA 243/75.

95 Selin, ”Lääketiede, huumeriippuvuus ja huumeriippuvuuden hoito Suomessa 1965–2005,” 255.

96 JSHA 107/75.

97 Ibid.

98 JSHA 8515.

99 On Fromm, see Petteri Pietikainen, *Alchemists of Human Nature: Psychological Utopianism in Gross, Jung, Reich and Fromm* (London: Routledge, 2015).

100 JSHA 1975.

101 JSHA 429/72.

102 JSHA 503/71.

103 Francisco Fernandez de Alba, *Sex, Drugs, and Fashion in 1970s Madrid* (Toronto: University of Toronto Press, 2020), 69–90.

104 JSHA N60/71.

105 JSHA 297/75.

106 JSHA 430/70.

107 JSHA 391/71.

108 JSHA 101/75.

109 In Finnish, *Suomen Kansan Demokraattinen Liitto*. The political position varied from left-wing to far-left, and by many, it was perceived as a communist front.

110 JSHA N82/72.

111 JSHA 484/71.

112 Salasuo, “Tajunnan vallankumous,” 106.

113 Stephens, *Germans on Drugs*, 182.

